# Biochemical Characterization of a Carrageenase, Car1383, Derived From Associated Bacteria of Antarctic Macroalgae

**DOI:** 10.3389/fmicb.2022.851182

**Published:** 2022-03-31

**Authors:** Jiang Li, Xiaoqian Gu, Qian Zhang, Liping Fu, Jiaojiao Tan, Luying Zhao

**Affiliations:** ^1^Key Laboratory of Ecological Environment Science and Technology, First Institute of Oceanography, Ministry of Natural Resources, Qingdao, China; ^2^CAS Key Laboratory of Experimental Marine Biology, Institute of Oceanology, Chinese Academy of Sciences, Qingdao, China

**Keywords:** carrageenase, Antarctic macroalgae, associated bacteria, metagenome, site-directed mutagenesis

## Abstract

A carrageenase gene, *car1383*, was obtained from the metagenome of Antarctic macroalgae-associated bacteria. The amino acid sequence of its product showed up to 33% similarity with other carrageenases and contained a GH16-family motif. The recombinant Car1383 was heterologously expressed in *Eschericia coli* and exhibited maximal activity at 50°C and pH 6.0, with a *K_m_* of 6.51 mg/ml and a *V_max_* of 55.77 U/mg. Its activity was enhanced by some cations (Na^+^, K^+^, and Fe^2+^), but inhibited or inactivated by others (Sr^2+^, Ca^2+^, Ni^2+^, Ba^2+^, Mn^2+^, Cu^2+^, Fe^3+^, and Mg^2+^). Car1383 degraded carrageenan into neocarrabiose and neocarratetraose. Site-directed mutagenesis indicated that putative active sites, E_190_ and E_195_, conserved sites, W_183_ and G_255_, play important roles in Car1383 activity. This study provides a new candidate for the industrial preparation of bioactive algal oligosaccharides.

## Introduction

Macroalgae contribute significantly to global primary production and have important industrial applications. Some species of red seaweeds (Rhodophyta) have cell walls that are mainly composed of carrageenan (Kloareg and Quatrano, 1988; Ruiter and Rudolph, 1997). Because carrageenan has gelling and emulsifying properties, it is used as a constituent of foods, personal care, and cosmetic products, and laboratory materials for microbiological research (Li et al., 2014). Notably, the oligosaccharides derived from carrageenan have pharmacological activities, so they are useful materials for drug research and development (Ghanbarzadeh et al., 2018). Oligo-carrageenans degraded by microbial enzymes can be more advantageous than those produced by acid hydrolysis, because enzymes react with their substrates in a mild way and generate oligo-derivatives with uniform molecular mass (Yao et al., 2014). Carrageenan-degrading enzymes are not only used to obtain bioactive carrageenan-derived oligosaccharides, but also to generate products for the textile and detergent industries, as saccharification agents for bioethanol production, and as a tool to prevent red algae blooms and reuse algal biomass (Martin et al., 2014; Chauhan and Saxena, 2016).

Despite the large body of research on bacterial carrageenases, few of them have been purified to homogeneity and most are not very well characterized (Antonopoulos et al., 2004). Carrageenase are of considerable interest, because much less is known about the enzymatic-degradation products of carrageenan than about those of other algal polymers such as agar and alginate (Michel et al., 2001a,b; Chauhan and Saxena, 2016). Therefore, identification of efficient microbial carrageenases for the production of bioactive algal oligosaccharides, especially from bacteria in special environments, is a growing field that may have a great impact in the coming years.

The Antarctic environment shows high species richness and has abundant endemic macroalgal species (Wulff et al., 2009). Antarctic macroalgae host complex and diverse microbial communities, which play an important role in carbon metabolism in the marine ecosystem of the Southern Ocean. In addition, it is likely that other marine organisms utilize the by-products of algal degradation. Moreover, those macroalgae-associated bacteria are an important source of novel and exploitable carbohydrate-active enzymes (Michel et al., 2006; Martin et al., 2014).

There is increasing interest in the discovery and diversity analysis of polysaccharide-degrading bacteria from the surface of Antarctic macroalgae. A recent study reported that an Antarctic marine red alga shelters fungi with carrageenolytic and agarolytic activities, as determined by enzymatic assays (Laura et al., 2018). However, to our knowledge, there are no previous reports on the biochemical and functional properties of carrageenases isolated from Antarctic bacteria.

Until the last decades, only culturable microorganisms that are naturally able to express the target enzyme were investigated as a source of natural enzymes. However, the purification of these organisms and their enzymes was expensive and time-consuming. Now, metagenomic approaches have made it much more efficient to identify the biodiversity of organisms and novel biocatalysts from many different environments, including the marine ecosystem (Berlemont et al., 2009). By using a metagenomic approach, several hundred novel CAZymes have been retrieved from metagenomes in the last decades (Li et al., 2009; André et al., 2014). However, recent genomic and meta-genomic data analyses suggest that there is still much to learn about the diversity and complexity of marine carbohydrate-degrading systems (Hehemann et al., 2014).

In present study, a carrageenase gene, *car1383*, was obtained from the metagenome of Antarctic macroalgae-associated bacteria, and then the biochemical characteristics of the recombinant Car1383 and its point mutations were investigated. The results of this study provide: (1) aκ-carrageenase gene sequence obtained from Antarctic strain for the CAZymes database; (2) a promising candidate for development of a recombinant industrial enzyme for the utilization of polysaccharides and macroalgae.

## Materials and Methods

### Sample Collection and Treatment

Six macroalgae samples, identified as *Callithamnion tetragonum*, *Plocamium cartilagineum*, *Pachymenia orbicularis*, *Desmarestia Antarctica*, *Phaeurus antarcticus*, and *Melanthalia abscissa*, were collected from the seashore of King George Island, Antarctica (Gui et al., 2020). After washing with sterilized seawater, the macroalgae samples were brought back to the laboratory to collect the surface-associated bacteria.

The macroalgae were again rinsed with sterilized seawater, and then cut into fragments. The pieces were placed in sterilized tubes, and then 20 ml sterilized seawater was added. The mixture was shaken for 30 s, three times, with a vortex mixer. The liquid was filtered to trap the associated bacteria on a filter membrane (47 mm × 0.22 μm, Merck Millipore, Darmstadt, Germany).

### DNA Extraction and Sequencing

The total genomic DNA of enriched macroalgae-associated bacteria was extracted using the FastDNA Spin Kit For Soil (MP Biomedicals, Santa Ana, CA, United States) following the manufacturer’s instructions. The quantified DNA samples were sequenced and analyzed on the Illumina HiSeq platform (Illumina, San Diego, CA, United States) by Novogene Bioinformatics Technology Co., Ltd (Beijing, China).

### Strain and Plasmid

The vector pUC118, restriction endonuclease and *Eschericia coli* DH5α cells were purchased from TaKaRa (Dalian, China), the expression vector pET-30a (+) and *E. coli* BL21 (DE3) cells were purchased from Novagen (Merck, United States). His-tag-specific purification kit was purchased from GE Healthcare (Pittsburgh, PA, United States). All the chemical reagents are chemically pure.

### Media and Dye Solution Compositions

The compositions of the media and Lugol’s dye solution were as follows: Luria–Bertani (LB) medium: tryptone 10.0 g/L, yeast powder 5.0 g/L, NaCl 10.0 g/L; Screening plates: tryptone 10.0 g/L, yeast powder 5.0 g/L, carrageenan 15 g/L, and NaCl 10.0 g/L. The LB and screening plate media were sterilized by autoclaving (1.21 × 10^5^ Pa, 20 min). Lugol’s solution consisted of 5 g I_2_ and 20 g KI dissolved in 100 ml sterilized distilled water.

### Sequence Analysis and Gene Expression

A presumptive carrageenase gene, designated as *car1383*, was obtained from the predicted functional genes in the metagenomic data obtained from Antarctic macroalgae-associated bacteria. Multiple sequence alignment was conducted using DNAMAN software.^1^ BlastP and BlastN^2^ and Motif Search^3^ were used for identity and motif analyses, respectively. A phylogenetic tree was conducted using the neighbor-joining (NJ) method by MEGA 5.0. The sequence of *car1383* with specific restriction sites of *Sal*I/*Xho*I was synthesized by the Beijing Genomics Corp (Beijing, China). This sequence was ligated to the pET30a vector, which was digested with same restriction endonucleases, to obtain the recombinant plasmid pET30a + *car1383*. The recombinant plasmid was used to transform to *E. coli* BL21, and the transformed cells were cultured on a screening plate on medium containing 50 μg/ml kanamycin. After culture for 24 h at 37°C, the bacteria were dyed with Lugol’s solution. The positively transformed strain showed a distinct clear circle and was assumed to have carrageenase activity.

To obtain purified recombinant carrageenase Car1383, the positive strain was inoculated into LB liquid medium containing 50 μg/ml kanamycin. When the OD_600_ of the culture medium reached 0.6, adding 0.5 mM isopropyl-β-D-thiogalactopyranoside (IPTG) to induce the expression of the fusion protein Car1383. The culture medium was centrifuged at 10,000 *g* at 4°C, then the pellet was dissolved in PBS buffer (pH 7.0). The harvested cells were disrupted using a high-pressure cell cracker (Constant Systems, Co., Ltd., Daventry, United Kingdom). After centrifugation, the supernatant was purified using a His Bind Purification kit (GE Healthcare, Piscataway, NJ, United States) according to the manufacturer’s instructions. The purified recombinant protein was identified by sodium dodecyl sulfate–polyacrylamide gel electrophoresis (SDS-PAGE).

### Enzyme Activity Assays

The enzyme activity of Car1383 was determined using the 3,5-dinitrosalicylic acid method (Miller, 1959). In brief, purified Car1383 was pre-incubated with 0.1% κ-carrageenan solution (0.2 mol/L Na_2_HPO_4_-NaH_2_PO_4_ buffer, pH 7.0) at 50°C for 30 min, and then the reaction was terminated by heating 100°C for 10 min. One unit of enzyme activity was defined as the amount of enzyme that catalyzes the conversion of 1 μmol of reducing sugars per minute under these conditions.

### Biochemical Characteristics of Recombinant Car1383

#### Temperature and Thermostability

For the optimal temperature assay, 1 ml Car1383 was pre-incubated with 1 ml 0.05% (M/V) κ-carrageenan solution (0.2 mol/L Na_2_HPO_4_-NaH_2_PO_4_ buffer, pH 7.0) in a temperature range of 20 ~ 80°C for 1 h. The enzyme activity was determined by the DNS method as described above, and the highest enzyme activity was defined as 100%.

To investigate the thermal stability of the enzyme, 1 ml Car1383 was incubated with 1 ml 0.1% κ-carrageenan solution (0.2 mol/L Na_2_HPO_4_-NaH_2_PO_4_ buffer, pH 7.0) at 30, 40, and 50°C for times ranging from 0 to 24 h, then the residual activity was determined by the DNS method. The activity of Car1383 pre-incubation with κ-carrageenan solution for 1 h at the corresponding reaction temperature was defined as 100%.

#### Determination of Optimal pH for Car1383 Activity

To determine the optimum pH for enzyme activity, 1 ml Car1383 was incubated with 1 ml 0.1% κ-carrageenan solution in different buffers: Na_2_HPO_4_-citrate buffer (pH 3.0–6.0), NaH_2_PO_4_-Na_2_HPO_4_ buffer (pH 6.0–8.0), Tris–HCl buffer (pH 8.0–9.0), and glycine-NaOH buffer (pH 9.0–11.0) at 50°C for 1 h. The enzyme activity was determined by the DNS method described above, and the highest enzyme activity was defined as 100%.

#### Effects of Metal Ions on Car1383 Activity

To determine the effects of metal ions on enzyme activity, Car1383 was pre-incubated with different metal cations (manganese, Mn^2+^; ferrous and ferric iron, Fe^2+^, Fe^3+^; nickel, Ni^2+^; calcium, Ca^2+^; strontium, Sr^2+^; magnesium, Mg^2+^; barium, Ba^2+^; cadmium, Cd^2+^, and copper, Cu^2+^), each at a concentration of 5 mM, for 30 min at 50°C and pH 6.0. Then, the residual enzyme activity of Car1383 was evaluated by the DNS method. The enzyme activity without metal ions was defined as 100%.

#### The Kinetic Parameters of Car1383

The initial reaction rate of Car1383 toward different concentrations of κ-carrageenan substrate (0.025, 0.050, 0.080, 0.100, 0.160, and 0.200 mg/ml) was assayed using the standard method described above. The values of the Michaelis–Menten constant (*K*_m_) and maximal reaction rate (*V*_max_) were calculated from the Lineweaver–Burk equation using GraphPad Prism 8 statistical package (GraphPad Software, Inc., United States; Morrison, 2002). All biochemical characterization assays were performed in triplicate.

### Analysis of Degradation Products

#### Thin Layer Chromatography

Purified Car1383 solution (10 μg/ml) was added to an equal volume of κ-carrageenan substrate solution (0.05%, M/V) and the reaction mixture was incubated at 50°C in NaH_2_PO_4_-Na_2_HPO_4_ buffer solution (pH 6.0) for 24 h. Subsequently, the supernatant of the reaction products was spotted onto a Silica Gel 60 F_254_ plate (Merck). The thin layer chromatography (TLC) plate was developed with *n*-butanol–acetic acid–water solution (1:2:1, V/V) for analysis of the enzymolysis products. The resultant oligosaccharides were detected by spraying with 10% (V/V) H_2_SO_4_ in ethanol solution, followed by heating at 80°C for 15 min.

#### Electrospray Mass Spectrometry Analysis

To identify the enzymolysis products further, the liquid chromatography with tandem mass spectrometry analysis was performed. The purified oligosaccharide solutions (1 mg/ml) were injected using a Rheodyne loop (5-μl), and used the 50% aqueous methanol as mobile phase with a flow rate of 200 μl/min. For the electrospray ion (ESI) source, the spray voltage was set at 4 kV, with a sheath gas (nitrogen) flow rate of 30 arbitrary units, an auxiliary gas (nitrogen) flow rate of 5 arbitrary units, a tube lens voltage of −250 V, a capillary temperature of 350°C, and a capillary voltage of −48 V. The scan parameter were as follows: rate, normal; type, full; microscan number, 3; and mass ranges, m/z 200–1,500.

### Mutation Analyses

**Figure 1:**
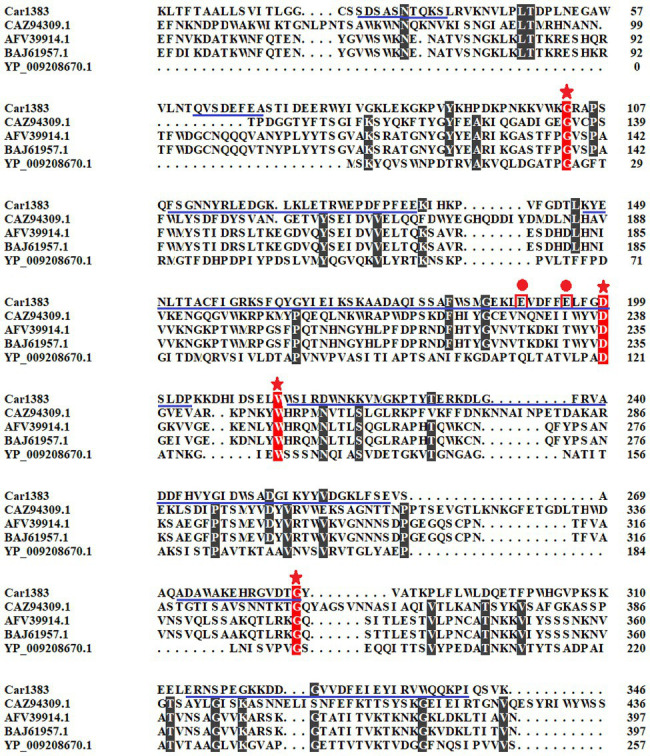
Multiple sequences alignment and motif analysis of Car1383. The conserved sites were indicated with stars. The predictive active residues for Car1383 were indicated with circle dots. The predictive motif of GH16 was highlighted with underlines.

To identify the active site responsible for the activity of Car1383, point mutations were introduced at two putative active sites and four conserved amino acids (Figure 1; Table 1). Mutated genes (M1–M18) corresponding to mutations listed in Table 1 were synthesized by the Beijing Genomics Corp (Beijing, China). These synthetic genes were first digested by restriction endonucleases *Sal*I/*Xho*I, then were purified by the Gel Extraction Kit. The purified gene was ligated into vector pET30a, which had been digested with the same two restriction endonucleases. These recombinant plasmid were transformed into *E. coli* BL21 (DE3) competent cells and confirmed by DNA sequencing. The transformants harboring recombinant plasmid were cultured in LB medium with 50 μg/ml kanamycin at 37°C to OD_600_ of 0.6 and induced with 0.5 mM isopropyl-β-D-thiogalactopyranoside (IPTG). Eighteen recombinant mutants were firstly checked using plate-based assays, then the enzymatic activity of purified recombinant proteins was determined using the standard DNS method described above.

**Table 1:** Mutational information of Car1383.

The active residues mutation				The conserved residues mutation			
Name	Mutants	Initial residue	Replaced residue	Name	Mutants	Initial residue	Replaced residue
M1	E159A	E_159_	A_159_	M7	G73K	G_73_	K_73_
				M8	D169K	D_169_	K_169_
M2	E165A	E_165_	A_165_	M9	W183K	W_183_	K_183_
				M10	G255K	G_255_	K_255_
M3	E159–165A	E_159_–E_165_	A_159_–A_165_	M11	G73R	G_73_	R_73_
				M12	D169R	D_169_	R_169_
M4	E159K	E_159_	K_159_	M13	W183R	W_183_	R_183_
				M14	G255R	G_255_	R_255_
M5	E165K	E_165_	K_165_	M15	G73H	G_73_	H_73_
				M16	D169H	D_169_	H_169_
M6	E159–165K	E_159_–E_165_	K_159_–K_165_	M17	W183H	W_183_	H_183_
				M18	G255H	G_255_	H_255_

*M1–M6: the mutational information of the active residues of Car1383. M7–M18: the mutational information of the conserved residues of Car1383. Initial residue was original amino acid in wild-type enzymes. Replaced residue was the amino acid which replaced the original amino acid at corresponding positions*.

### Statistical Analysis

The significant differences of enzyme activity between selected mutants were determined using one-way ANOVA with the SPSS 19.0 statistical software (^*^p < 0.05, ^**^p < 0.01).

## Results and Discussion

The presumptive kappa-carrageenase Car1383 obtained from metagenomics data of macroalgae-associated bacteria had a coding sequence of 1,041 nucleotides and encoded a protein of 346 amino acids. There are 22 amino acid residues as a potential signal sequence at the amino-terminal end of the encoded Car1383 protein analyzed by SignalP 4.1 server.^4^ Motif search analyses revealed a GH16 domain and two putative activity sites, E_190_ and E_195_. In Blast analyses, the deduced amino acid sequence of Car1383 showed 23.13% similarity with the kappa-carrageenase (CAZ94309.1) from *Zobellia galactanivorans*, 25.51% similarity with the kappa-carrageenase (AFV39914.1) from *Pseudoalteromonas* sp. QY203, 25.82% similarity with the kappa-carrageenase (BAJ61957.1) from *Pseudoalteromonas tetraodonis*, and 33.33% similarity with the kappa-carrageenase (YP_009208670.1) from *Achromobacter phage phiAxp-*3 (Figure 1).

**Figure 2:**
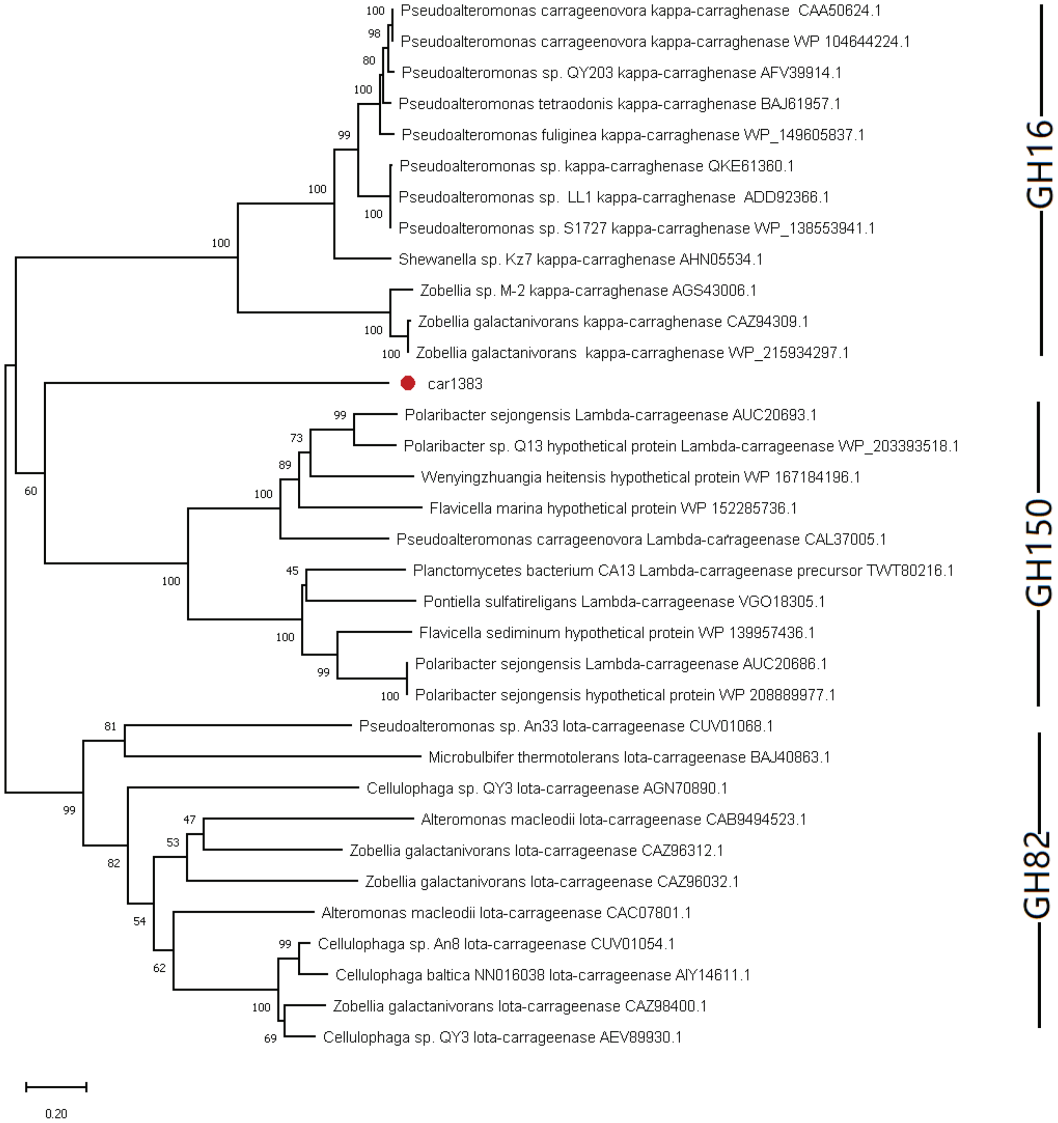
Phylogenetic analysis of Car1383 with other carrageenases based on the amino acid sequences. The tree was constructed by the neighbor-joining method. Bootstrap values (expressed as percentages of 2,000 replications) are shown at branching points.

It is known that there are three glycoside hydrolase (GH) families in carrageenase classified based on their amino acid sequence similarities: GH16 (κ-carrageenase), GH82 (ι-carrageenase), and GH150 (λ-carrageenase). The phylogenetic analysis based on amino acid sequence of carrageenases showed that Car1383 has far evolutionary relationship with any known GH families (Figure 2). Together with the motif analysis (Figure 1) and substrate specificity assay (Supplementary Figure S1), we deduced that Car1383 is a κ-carrageenase member belonged to GH16 family.

Algae-associated microbes are a major resource for the discovery of novel specific enzymes and secondary metabolites (Martin et al., 2014). However, little is known about the polysaccharides hydrolyzed by bacteria living on the surface of Antarctic marine macroalgae. Our results provide a sequence of GH16 family member, derived from Antarctic macroalgae-associated bacteria, to supplement CAZymes data.

### Expression and Purification of Car1383

**Figure 3:**
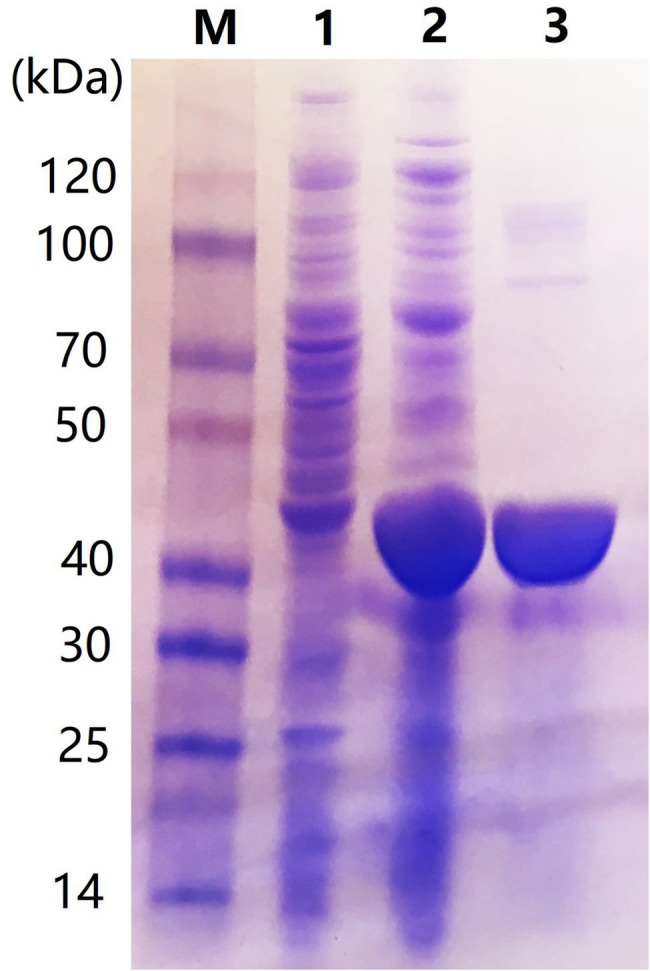
Sodium dodecyl sulfate–polyacrylamide gel electrophoresis (SDS-PAGE) of purified recombinant Car1383. Lane M: Protein markers (BioRad, Hercules, CA, United States); Lane 1: supernatant of recombinant *Eschericia coli* BL21 (DE3) induced by IPTG for 16 h at 16°C; Lane 2: pellet fraction of recombinant *E. coli* BL21 (DE3) induced by IPTG for 16 h at 16°C; and Lane 3: purified Car1383.

The *car1383* gene, encoding a mature form of the protein with 22 amino acids signal peptide sequence, was ligated to the pET30a vector and correctly expressed in *E. coli* BL21 (DE3). The SDS-PAGE result confirmed that 0.5 mM IPTG induced its expression. After purification using a Ni-NTA His Tag Kit, a single band corresponding to 40 kDa protein marker was obtained, as determined by SDS-PAGE. This size was consistent with the theoretical molecular mass of Car1383 (Figure 3).

### Biochemical Characterization of Car1383

**Figure 4:**
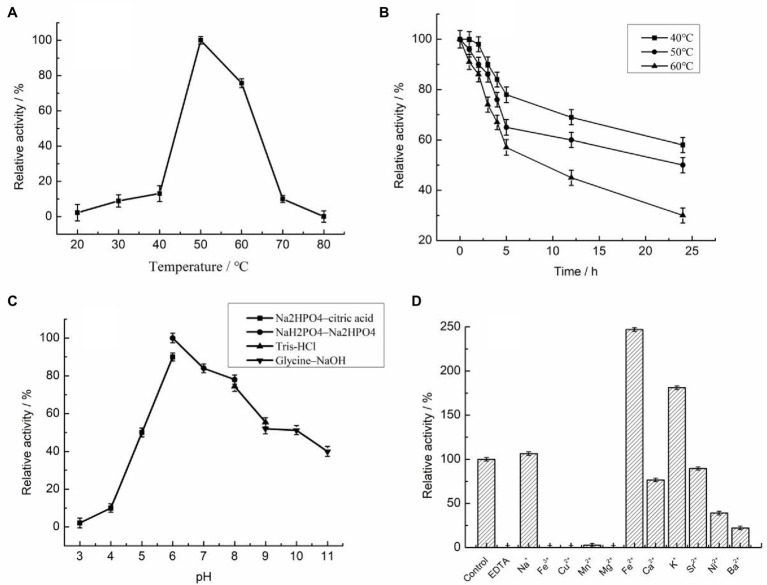
Biochemical properties of purified recombinant Car1383 (see Materials and Methods for details of purification and standard assay procedure). **(A)** Effect of temperature on Car1383 activity. **(B)** Thermostability of recombinant Car1383 at different temperatures. **(C)** Effect of pH on Car1383 activity determined at 50°C using different buffer: Na_2_HPO_4_–citric acid (pH 3.0–6.0), Na_2_HPO_4_–NaH_2_PO_4_ (pH 6.0–8.0), Tris–HCl (pH 8.0–9.0), or glycine–NaOH (pH 9.0–11.0). **(D)** Effects of metal salts (CaCl_2_, CuSO_4_) and chelators on Car1383 activity.

The reaction temperature and thermostability are very important biochemical characteristics of an enzyme for industrial applications. Unexpectedly, the optimal temperature for Car1383 activity was 50°C (Figure 4A), higher than those of CgkX, CgkS, CgkHC4, and CgkZ (Sun et al., 2010; Li et al., 2013; Liu et al., 2013; Wang et al., 2015; Table 2). Even though Car1383 originates from the Antarctic marine ecosystem, it maintained more than 70% of its initial activity at 60°C (Figure 4B). Thus, Car1383 has high thermostability, with half-lives of 9 h at 60°C and 24 h at 50°C. Carrageenan is highly viscous at room temperature and is an inhibitor of carrageenase degradation activity. Usually, increasing the dissolution temperature is used to reduce the viscosity of carrageenan solution. However, most identified carrageenases are unstable above 40°C (Chauhan and Saxena, 2016). Therefore, a thermostable carrageenase such as Car1383 is of considerable interest as a new biocatalyst for producing enzymatic-degradation products of carrageenan on a commercial scale.

**Table 2:** Biochemical characteristics of reported κ-carrageenases.

Name	Origin	Temp. optima (**°**C)	pH optima	*K*_m_ (mg/ml)	Product	References
Car1383	Macrogenome	50	6.0	6.51	2, 4, 6	This study
CgkAJ5	*Pseudoalteromonas* sp.	55	8.0	59.8	2,4,6,8,10	Morrison, 2002
CgkX	*Pseudoalteromonas* sp. QY203	45	7.2	-	2	Michel et al., 2001a
Cgk-K142	*Pseudoalteromonas tetraodonis*	55	8.8	-	-	Ruiter and Rudolph, 1997
CgkS	*Shewanella* sp. Kz7	45	8.0	716.8	2,4	Michel et al., 2001b
CgkHC4	*Tamlana* sp. HC4	30	8.0	7.63	2	Michel et al., 2006
CgkZ	*Zobellia* sp. ZM-2	39	6.0–8.0	0.84	4,6	Miller, 1959

The optimal pH for Car1383 activity was 6.0, lower than that reported for most other κ-carrageenases (Ma et al., 2010; Sun et al., 2010; Kobayashi et al., 2012; Li et al., 2013; Wang et al., 2015; Table 2). In our experiments, Car1383 retained almost 50% of its original activity at pH 10.0 and 40% of its original activity at pH 11.0 (Figure 4C).

Metal ions had different effects on the activity of recombinant Car1383. Its activity was partially inhibited by Sr^2+^, Ca^2+^, Ni^2+^, Ba^2+^, and Mn^2+^, and completely inhibited by Cu^2+^, Fe^3+^, and Mg^2+^. The activity of many enzymes is negatively affected by heavy metal ions like mercury (Hg^2+^), cobalt (Co^2+^), zinc (Zn^2+^), Cu^2+^, aluminum (A1^3+^), and lead (Pb^2+^), indicating that enzyme activity can be cation-independent and that these metals can alter the conformation of enzymes (Araki et al., 1999; Ma et al., 2010; Li et al., 2013). However, the carrageenase activity of recombinant Car1383 was enhanced by Na^+^, K^+^, and especially Fe^2+^, which more than doubled its initial activity. In other studies, cations such as sodium (Na^+^) and potassium (K^+^) were found to increase carrageenase activity (Ma et al., 2010; Li et al., 2013, 2014), especially high concentrations Na^+^ (Ma et al., 2013), while reagents such as iodoacetic acid, Tween-80, and EDTA had little effect on carrageenase activity (Sarwar et al., 1983). In our research, Car1383 activity was completely inhibited by EDTA (Figure 4D), indicating that metal ions play an important role in the activity of this enzyme.

### Enzymatic Kinetics Parameters

**Figure 5:**
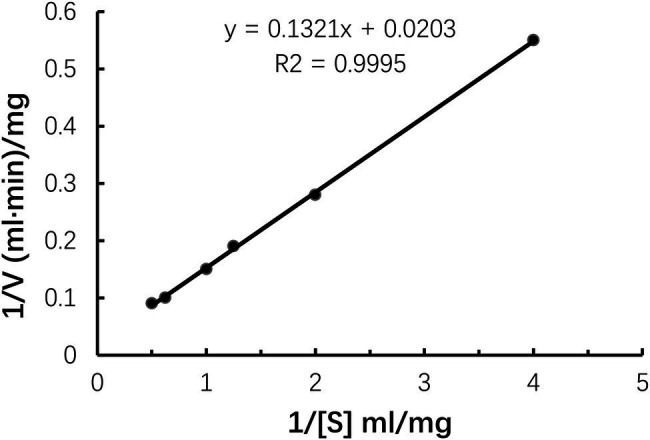
Kinetic parameters of purified recombinant Car1383 with different concentrations of κ-carrageenan substrate (from 0.01 to 0.1 g/L).

In this study, the *V*_max_ and *K*_m_ of Car1383 were 55.77 mg/ml·min and 6.51 mg/ml, respectively (Figure 5). The *K*_m_ of Car1383 was lower than that of some κ-carrageenases, such as CgkAJ5, CgkHC4, and CgkS (Ma et al., 2010; Sun et al., 2010; Wang et al., 2015), but higher than that of CgkZ (0.84 mg/ml; Liu et al., 2013; Table 2). The lower the *K*_m_ value for an enzyme, the higher its affinity for the given substrate. Therefore, the low *K*_m_ of Car1383 is indicative of a high affinity toward its substrate.

### Degradation Product Analysis

**Figure 6:**
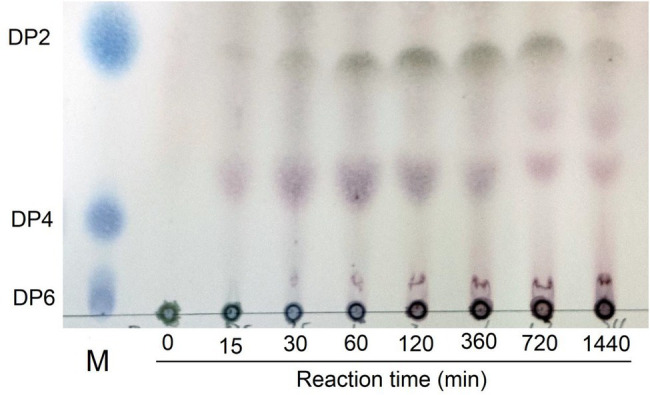
Thin-layer chromatogram of degradation products by recombinant Car1383. See Materials and Methods for details of standard assay conditions. a. Lane M: standard samples of neocarraobiose (Dp2), neocarraotetraose (Dp4), and neocarraohexaose (Dp6), respectively; Lane 0: Control, κ-carrageenan without Car1383 incubated for 60 min; Lane 15–1,440: Car1383 incubated with purified Car1383 for different reaction times (min).

Thin layer chromatography is a convenient and effective method to detect different polymerization degrees oligosaccharides (Dhalwal et al., 2008). From the TLC results, we observed that Car1383 degraded κ-carrageenan into different polymerization degrees oligosaccharides. According to the migration rate of degraded oligosaccharides, the main hydrolysis products were disaccharides (Dp2) and tetrasaccharides (Dp4). This range of products is the same as that produced by CgkS (Wang et al., 2015), but different from those produced by CgkAJ5, CgkX, CgkHC4, and CgkZ (Ma et al., 2010; Sun et al., 2010; Li et al., 2013; Liu et al., 2013; Table 2). Hexasaccharides (Dp6) and those with higher degrees of polymerization were not observed on the TLC plate (Figure 6). From 30 min to 12 h of hydrolysis, the Dp4 products became less abundant, while the Dp2 products became more abundant. This may mean that κ-carrageenan was firstly degraded into Dp6, then Dp6 was degraded into Dp2 and Dp4.

**Figure 7:**
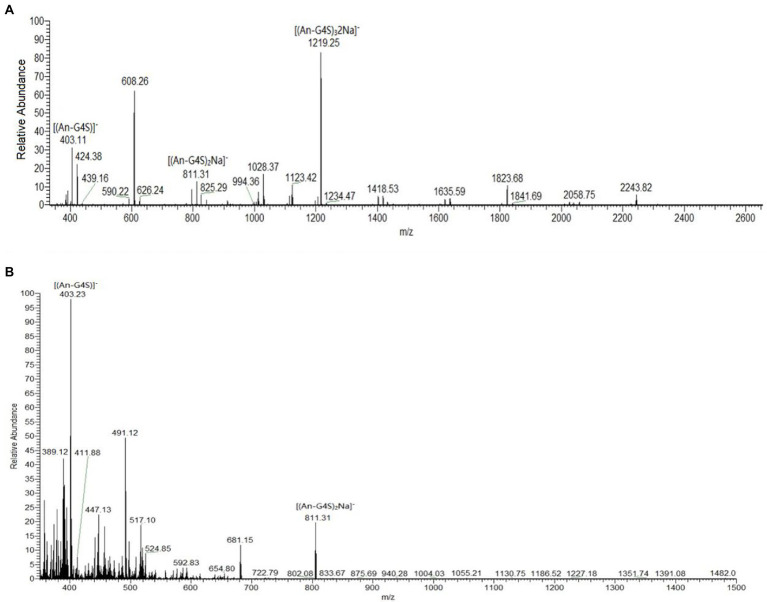
Results of electrospray mass spectrometry (ESI-MS) analyses of products of κ-carrageenan hydrolysis by purified Car1383. **(A)** for 30 min; **(B)** for 12h.

However, the presence of sulfate groups in sulfate oligosaccharides has an effect on migration and resolution of these oligosaccharides on TLC plates (Zhang et al., 2007), so, the degradation products were further analyzed by electrospray mass spectrometry (ESI-MS). The mass spectra of products after 30 min of hydrolysis had signals of κ-carrageenan oligosaccharides, mainly Dp2, Dp4, and Dp6. The peak at m/z 403.11 was assigned to a disaccharide [(G4S-An)]^−^ carrying one sulfate group. The peak at m/z 811.31 was assigned to a sodium salt of tetrasaccharide [(G4S-An)_2_Na]^−^ carrying two sulfate groups. And the peak at m/z 1219.25 was likely corresponding to a hexasaccharide with three sulfate groups carrying two sodium ions [(G4S-An)_3_2Na]^−^ (Figure 7A). In the mass spectra of the reaction mixture after 12 h of hydrolysis, only signals for Dp2 (m/z 403.23) and Dp4 (m/z 811.31) were observed, and there were no signals corresponding to Dp6 (Figure 7B). Together with the TLC results, the ESI-MS data indicated that κ-carrageenan was firstly degraded into Dp6, then Dp6 was degraded into Dp2 and Dp4. These results showed enzymatic properties of Car1383, for which the cleavage group was An-G4S unit, resulting in a series of neocarrabiose oligosaccharides with the code of An-G4S. Previous studies have shown that carrageenases cleave the internal β-1, 4 linkage in κ-, ι-, and λ-carrageenan (Shen et al., 2017), similar with reported carrageenases, Car1383 is an endohydrolases and belonged to the GH family.

### Single Point Mutation Analyses

**Figure 8:**
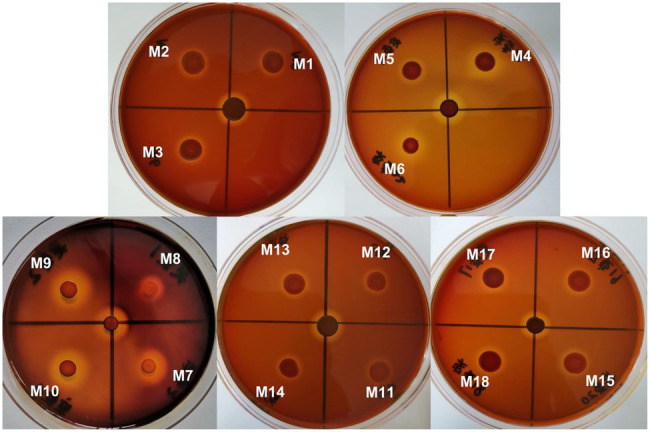
Plate-active results of different mutations. M1–M18 correspond to mutations listed in Table 1.

**Figure 9:**
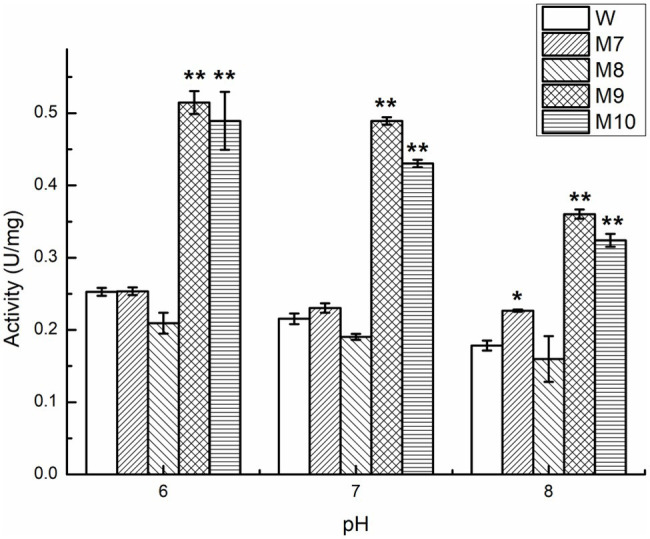
Comparison of enzyme activity of Car1383 and its variants at pH 6.0–8.0. *p < 0.05, **p < 0.01.

Protein engineering, including directed evolution and rational design, is an effective technology for improving enzyme properties (Liu et al., 2017). To understand the potential active sites responsible for activity, the residues E_190_ and E_195_ were replaced with Ala or Lys. To explore the effect of conserved sites on activity, four amino acid residues were replaced with alkaline amino acids, Lys, Arg, or His. The results showed that the activity of those mutants was lower than that of wild-type Car1383, whether E_190_ and E_195_ were replaced by Ala or Lys, or both of them were replaced with Ala or Lys. These findings suggested that E_190_ and E_195_ play important roles in the enzymatic activity of Car1383. In contrast, the activities of the mutants W183K and G255K were almost 2-fold higher than that of wild-type Car1383 (Figures 8, 9). This may be because Lys has a positively charged side chain, which leads to enhanced binding to negatively charged carboxyl groups of the substrate (Xu et al., 2021). However, the activity of the mutant D169K was a little lower than that of wild-type Car1383, maybe because the site was located near the active sites. The substitutions had little effect on the pH adaptability of the mutants W183K and G255K, which showed an optimal pH of 6.0 (Figure 8). Xu et al. (2021) successfully increased the catalytic efficiency of alginate lyase AlgL-CD through site-directed mutagenesis, and the catalytic activity of XynB (Wu et al., 2017) and alginate lyase AlgL (Jang et al., 2016) have been enhanced by rational design. Wu et al. (2017) reported that the substrate affinity of polysaccharide lyases could be improved by tight binding to Ca^2+^ *via* one amino acid substitution. Our previous site-directed mutagenesis study indicated that two Ca-binding sites are responsible for the Ca^2+^ activation and thermostability of agarase Aga3463 (Li et al., 2019).

In summary, we obtained a carrageenase Car1383, belonging to the GH16 family, derived from the metagenome of bacteria associated with the surface of Antarctic macroalgae. Our results showed that recombinant Car1383 has a higher optimal reaction temperature and better thermostability than most reported carrageenases, even though it is derived from the Antarctic environment. This study provides a new candidate for industrial preparation of bioactive algal oligosaccharides and paves the way for efficient utilization of macroalgae.

## Data Availability Statement

Publicly available datasets were analyzed in this study. The data presented in the study are deposited in the GenBank repository, accession number OK300048.

## Author Contributions

JL designed the research, supervised the project, and wrote the manuscript. XG, QZ, LF, and LZ performed the experiments. JT analyzed the MS data. All authors contributed to the article and approved the submitted version.

## Funding

This work was supported by Impact and Response of Antarctic Seas to Climate Change (RFSOCC2020-2022).

## Conflict of Interest

The authors declare that the research was conducted in the absence of any commercial or financial relationships that could be construed as a potential conflict of interest.

## Publisher’s Note

All claims expressed in this article are solely those of the authors and do not necessarily represent those of their affiliated organizations, or those of the publisher, the editors and the reviewers. Any product that may be evaluated in this article, or claim that may be made by its manufacturer, is not guaranteed or endorsed by the publisher.

## Footnotes

^1^ http://www.lynnon.com/

^2^ http://blast.ncbi.nlm.nih.gov/Blast.cgi

^3^ http://www.genome.jp/tools/motif/

^4^ http://www.cbs.dtu.dk/services/SignalP/
