# Supplementary material for: Biochemical Characterization of a Carrageenase, Car1383, Derived From Associated Bacteria of Antarctic Macroalgae
Source: Front Microbiol. 2022 Mar 31;13:851182. doi: 10.3389/fmicb.2022.851182 (PMC9009511; doi:10.3389/fmicb.2022.851182)
Supplement: Supplementary file 1 [file Table_1.DOC]

Supplementary Figures

#
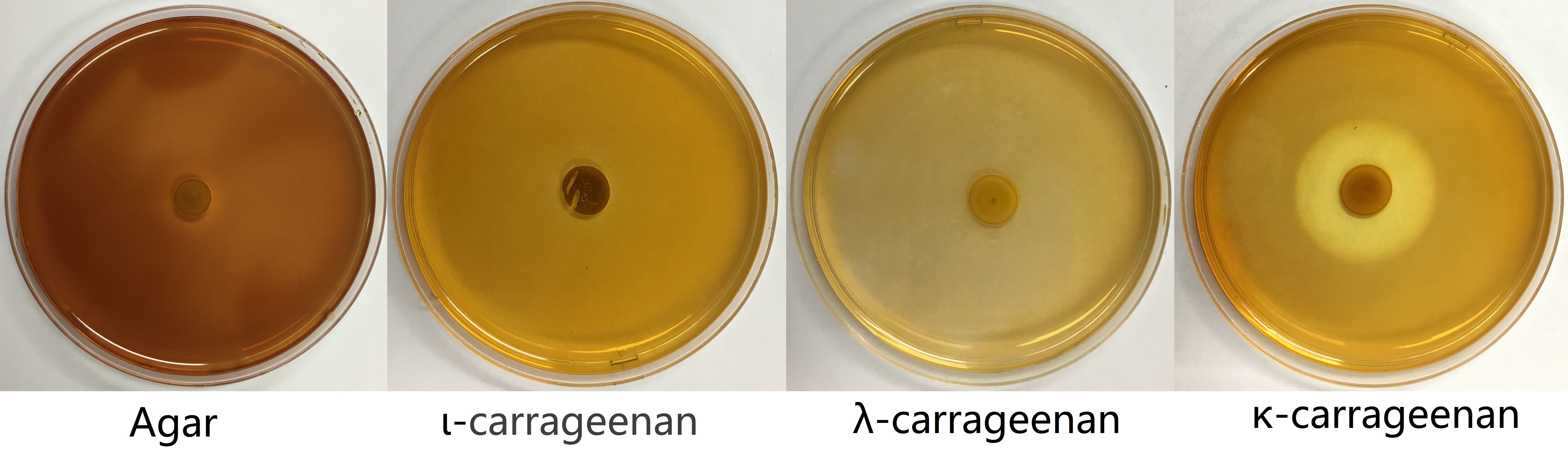


**Supplementary Figure 1.** The substrate specificity assay of recombinant Car1383

Lugol’s solution staining showed distinct clear zone around the clone of recombinant Car1383on the κ-carrageenan plate, but not on Agar, ι-carrageenan and λ-carrageenan plates.
